# Gene transfer to pre-hematopoietic and committed hematopoietic precursors in the early mouse Yolk Sac: a comparative study between *in situ *electroporation and retroviral transduction

**DOI:** 10.1186/1471-213X-7-79

**Published:** 2007-07-02

**Authors:** Sébastien JD Giroux, Celmar Alves-Leiva, Yann Lécluse, Patrick Martin, Olivier Albagli, Isabelle Godin

**Affiliations:** 1 INSERM U790, Institut Gustave Roussy-PR1; 39, Rue Camille Desmoulins, 94805 Villejuif, France; 2 Institut Gustave Roussy, 39, Rue Camille Desmoulins; 94805 Villejuif, France; 3 Université de Paris XI, Orsay, France; 4 CNRS UMR6548; Université Nice-Sophia Antipolis, Bât. Sciences Naturelles; Parc Valrose, 06108 Nice Cedex 2; France

## Abstract

**Background:**

Hematopoietic development in vertebrate embryos results from the sequential contribution of two pools of precursors independently generated. While intra-embryonic precursors harbour the features of hematopoietic stem cells (HSC), precursors formed earlier in the yolk sac (YS) display limited differentiation and self-renewal potentials. The mechanisms leading to the generation of the precursors in both sites are still largely unknown, as are the molecular basis underlying their different potential. A possible approach to assess the role of candidate genes is to transfer or modulate their expression/activity in both sites. We thus designed and compared transduction protocols to target either native extra-embryonic precursors, or hematopoietic precursors.

**Results:**

One transduction protocol involves transient modification of gene expression through *in situ *electroporation of the prospective blood islands, which allows the evolution of transfected mesodermal cells in their "normal" environment, upon organ culture. Following *in situ *electroporation of a GFP reporter construct into the YS cavity of embryos at post-streak (mesodermal/pre-hematopoietic precursors) or early somite (hematopoietic precursors) stages, high GFP expression levels as well as a good preservation of cell viability is observed in YS explants. Moreover, the erythro-myeloid progeny typical of the YS arises from GFP^+ ^mesodermal cells or hematopoietic precursors, even if the number of targeted precursors is low. The second approach, based on retroviral transduction allows a very efficient transduction of large precursor numbers, but may only be used to target 8 dpc YS hematopoietic precursors. Again, transduced cells generate a progeny quantitatively and qualitatively similar to that of control YS.

**Conclusion:**

We thus provide two protocols whose combination may allow a thorough study of both early and late events of hematopoietic development in the murine YS. *In situ *electroporation constitutes the only possible gene transfer method to transduce mesodermal/pre-hematopoietic precursors and analyze the earliest steps of hematopoietic development. Both *in situ *electroporation and retroviral transduction may be used to target early hematopoietic precursors, but the latter appears more convenient if a large pool of stably transduced cells is required. We discuss the assets and limitation of both methods, which may be alternatively chosen depending on scientific constraints.

## Background

In the mouse embryo, hematopoiesis involves the sequential contribution of two independent waves of precursors (for review, see [1]). The first precursor generation occurs in the extra-embryonic compartment, where the yolk sac (YS) blood islands produce erythro-myeloid-restricted precursors, from 7.5 days post-coitus (dpc). The second generation of hematopoietic precursors takes place, from 8.5 to 11.5 dpc, in the region comprising the Aorta and underlying mesenchyme, which is referred to first as para-aortic splanchnopleura (P-Sp: 8.5–10 dpc), then as aorta-gonads-mesonephros region (AGM: 10–11.5 dpc). The P-Sp/AGM produces hematopoietic precursors endowed with the multilineage differentiation potential and long term reconstitution (LTR) capacity that characterise Hematopoietic Stem Cells (HSC). The P-Sp/AGM prospective territory, the caudal intra-embryonic splanchnopleura (Sp: before 8.5 dpc) does not harbour a hematopoietic potential when cultured *in vitro *on OP9 stromal cells [2]. Yet, when the early Sp is cultured *in toto *for a few days, it now produces hematopoietic precursors, which harbour a multilineage differentiation potential as well as LTR capacity [3,4]. As the Sp are explanted before the establishment of vascular connection with the extra-embryonic vascular network, which occurs at the 4–5 somite-stage [5,6], this observation shows that the intra-embryonic site is capable of autonomously generating HSC. In contrast, the progeny of similarly treated YS lacks both multipotentiality (they are unable to generate lymphoid cells) and LTR activity (they only provide short term erythro-myeloid reconstitution of irradiated recipients) [5,6].

The analysis of gene function during the successive steps leading to the emergence, in the YS, of hematopoietic precursors with limited differentiation and self-renewal potentials, by comparison to the events occurring in the intra-embryonic hemogenic site, constitutes a major issue for understanding the process leading to HSC generation. Indeed, the inability of extra-embryonic precursors to provide a lymphoid progeny and to maintain in the long term might result from a divergence in the pathways leading from mesodermal cells to a committed hematopoietic progeny. This divergence may be (potentially) reflected by differential gene expression at the time of hematopoietic induction in both hemogenic sites. We aim to assess whether genes differentially expressed in the extra- and intra-embryonic hemogenic sites do indeed play a role in the distinct potentials displayed by extra- and intra-embryonic precursors through the forced expression in the YS of genes only expressed in the intra-embryonic Sp.

Most of the investigations aimed to characterize hematopoietic development pathways in the mouse YS involve the analysis of either genetically engineered mice strains or *in vitro *differentiation potential of modified ES cells, which may be efficiently transduced by "classical" electroporation procedure or retroviral transduction [7]. We here describe and test an alternative approach that might help to decipher early steps of developmental hematopoiesis, by directly targeting YS cells. Moreover, to be able to separate potential effects on the emergence of native hematopoietic precursors from the blood islands mesoderm from effects on further differentiation and maintenance of these precursors, we designed and compared two independent protocols. The first, aimed to transiently transduce early blood islands mesodermal/pre-hematopoietic cells involves *in situ *electroporation, followed by an organ culture step prior to hematopoietic development investigations. The second, designed to target YS-hematopoietic precursors at later stages is based on retroviral transduction.

Embryo transduction through *in situ *electroporation [8], initially used mainly to analyse avian nervous system development, is now frequently used to study early stages of mouse ontogeny. This approach allows the introduction of transgenes into embryonic tissues explants [9] or into cells of the gastrulating mouse embryo [10-12] to follow cell fate, cell migration and lineage differentiation. We show here that this technique, which permits a controlled spatio-temporal delivery of transgenes and is compatible with the maintenance of transduced cells in their "natural" environment, is appropriate to analyze YS-blood islands mesodermal/pre-hematopoietic cells.

Retroviral transduction also constitutes an efficient approach to transfer cDNA into hematopoietic cells as well as HSC. Although efficiently used to transduce ES cells [7], it has yet been rarely used to transduce cells from early embryos. To our knowledge, it has only been used once to target 8 dpc YS hematopoietic precursors [13]. In this study, hematopoietic precursors were shown to acquire long-term multilineage hematopoietic reconstitution of irradiated recipients upon stable HoxB4 expression. However, the extent of efficiency and versatility of this method on YS cells was not reported. Here, it is shown that retrovirus-mediated gene transfer is inefficient to transduce YS mesodermal/pre-hematopoietic precursors, but is a powerful method to genetically modify hematopoietic precursors from YS at later stages.

## Results

### A-Gene transfer into early precursors from 7 dpc YS-blood islands

#### I-Determination of suitable stages for mesodermal/pre-hematopoietic cell targeting

To precisely identify the stage when blood islands are enriched in immature mesodermal or pre-hematopoietic cells, we used lmo2 mRNA expression as a marker of immature blood islands precursors. Indeed, the LIM-zinc finger transcriptional co-regulator lmo2 is required for the generation of hematopoietic precursors of both extra- and intra-embryonic origin [14,15]. Lmo2 is expressed in the extra-embryonic mesoderm that gives rise to hematopoietic precursors [16,17], but also by their erythroid progeny. The differentiation of blood islands mesoderm into mature erythroid cells occurs extremely fast. We thus used β-H1 mRNA expression, as the earliest marker of differentiating erythroid cells, namely primitive erythroblasts. We compared the temporal evolution of the expression of both markers, during early blood islands formation (7.25 to 8 dpc), to select the time window when lmo2 is expressed and β-H1 not yet/poorly expressed.

**Figure 1:**
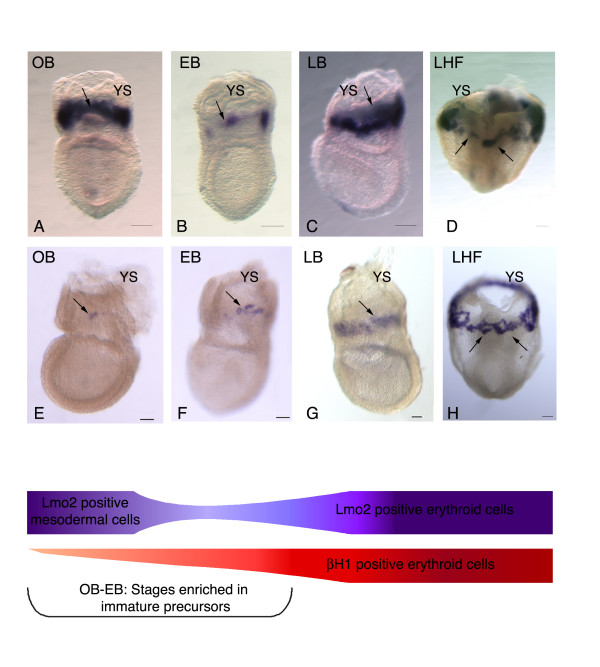
**Determination of the development stages enriched in immature precursors**. Upper panel: Temporal evolution of lmo2 expression from the OB to LHF stages: initially expressed by most extra-embryonic mesodermal cells (A), lmo2 expression rapidly restricts to a smaller cell number at the EB stage (B). Lmo2 expression subsequently expands with the development of blood islands endothelial and hematopoietic cells from LB (C) to LHF (D), and subsequent stages. Middle panel: Transcripts of the embryonic globin β-H1 are present in a minute cell number at the OB/EB stages (E, F). During the following stages (LB: G to LHF: H, and subsequent stages), β-H1 expressing erythroid cells rapidly expand. The arrows in the upper and middle panels point to equivalent zone of the blood islands. Lower panel: The comparative evolution of lmo2 and β-H1 expressions points to embryos at the OB/EB stages as enriched in immature precursors. Scale bar: 100 μm.

Lmo2 transcripts are first detected at the OB stage in the whole extra-embryonic mesoderm (Fig. 1A) and are subsequently restricted, at the EB stage (Fig. 1B), to a subset of mesodermal cells, which might correspond to blood islands precursors. Upon proliferation of these precursors, lmo2 expression expends to encompass all hematopoietic derivatives of the blood islands, from the LB to LHF stages (Fig. 1C, D) [17].

The first β-H1 transcripts are detected at the OB stage in a limited number of cells located within the lmo2-expressing ring (Fig. 1E). The number of cell clusters expressing β-H1 gradually increases, leading to the formation, at the LB stage, of a ring which expression overlaps that of lmo2 (Fig. 1F, G). During the following stages (EHF/LHF), this correlation of lmo2 and β-H1 expression is maintained (Fig. 1H). The correlation of these two expression patterns strongly suggest that mesodermal and/or pre-hematopoietic precursors are enriched in the OB to EB YS blood islands, even if mesodermal cells and native hematopoietic precursors may be still present in the blood islands at later stages. Targeting mesodermal cells was thus conducted at the OB to EB stages of development (Fig. 1 Lower panel).

#### II-Developmental constraints imposed on the choice of methodology

At these developmental stages, the determination and further proliferation/differentiation of hematopoietic precursors strictly depend on endoderm/mesoderm interaction. This implies that a method allowing the maintenance of tissue structure during transduction will be required for suitable development of a hematopoietic progeny from transduced cells. Accordingly, when we nevertheless tested the feasibility of dissociated cells transduction at the OB-EB stages using lipofection (using ExGen500) or retroviral transduction (see paragraph B2), we found that, in both instance, the transduction levels were naught, and cell viability drastically affected (Data not shown).

#### III-Viral-mediated transduction and in situ electroporation of 7dpc YS-blood islands

Given these constraints, two alternative approaches could be undertaken to transfer genes into the YS while keeping its tri-dimensional structure, viral-mediated transduction [18] and *in situ *electroporation. Indeed, both methods have already been used to transfer genes in other embryonic tissues and stages.

We first tested the ability of dissected YS to be transduced *in toto *by exposure to retroviral supernatant. OB-EB YS-explants were cultured in medium containing viral particles for 24 hours (for the method, see B). Explants were subsequently cultured in fresh medium for two days before assessing GFP levels and hematopoietic cell production. At the outset of the organ culture step, the morphology of transduced YS appeared affected by the process. Cytometry analysis of these YS explants showed that only 0.1% of viable cells expressed GFP. These cells were in addition unable to produce a hematopoietic progeny (data not shown).

We thus investigated *in situ *electroporation as a second approach.

##### 1-General scheme of the *in situ *electroporation protocol

To optimize this method, we used the peGFP-C1 plasmid (Clontech), which brings on a ubiquitous expression of the GFP reporter gene driven by the CMV promoter. After injection of the plasmid into the YS cavity and *in situ *electroporation, the YS is dissected from the embryo and kept in organ culture for three days.

The evolution of electroporated YS is systematically compared to non-electroporated control and/or control YS electroporated without plasmid. The effect of *in situ *electroporation on the development and viability of YS-explants is assessed through morphology examination after the organ culture step, as well as by the detection of endothelial and erythroid cells differentiation within the explants. GFP levels allow the monitoring of transduction efficiency. Finally, the characterization of the hematopoietic progeny of GFP^+ ^sorted cells, obtained upon culture on OP9 stromal cells, which normally allows the hematopoietic differentiation of mesodermal precursors [19], is used to typify the targeted precursors.

##### 2-*In situ *electroporation parameters adapted to YS-mesoderm transduction

Previous studies involving *in situ *electroporation stressed the importance of several parameters for an efficient DNA delivery as well a proper preservation of cell viability, and an accurate tissue targeting [20,21].

The electroporation procedure (voltage (V), pulse duration (ms), number of pulses applied and intervals (ms) between two pulses) is critical for the maintenance of tissue integrity and effectiveness of construct delivery.

The choice of the electrode type used, the distance between them and their position regarding the injected site clearly constitute the most important parameters influencing cell targeting.

###### a-Plasmid delivery

**Figure 2:**
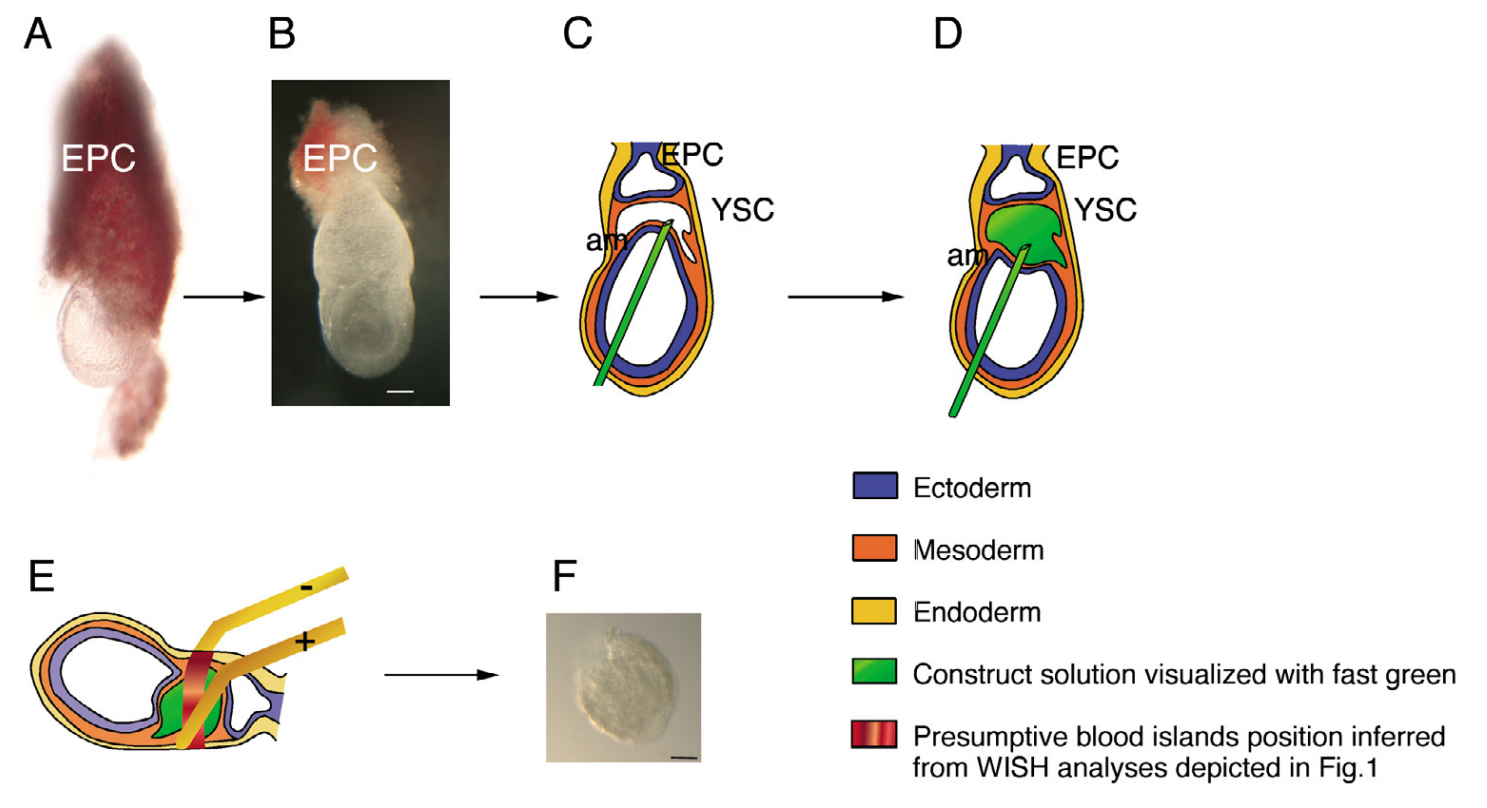
**Plasmid injection protocol**. Abbreviations: am: amnion; EPC: Ectoplacental cone; YSC: YS cavity OB-EB stage embryos are dissected from the decidua (A). The Reichert membrane is removed, while the EPC is kept in place (B). The plasmid solution is injected into the YSC, from the node region through the amnion (C). Plasmid filling of the YSC is visualised with fast green (D). Prior to pulse application, the electrodes are positioned parallel to the prospective blood islands ring (E). After electroporation, the YS is dissected from the embryo and placed in organ culture (D). Scale bar: 100 μm.

Targeting the YS-mesodermal/pre-hematopoietic precursors, while avoiding endoderm transduction, requires the injection of the construct into the YS cavity, since the mesoderm layer is facing this cavity while endoderm is exposed to the external environment. The embryos are freed from the decidua and Reichert's membrane (Fig. 2A, B) to allow injection. A capillary, back-filled with 1 μg/μl *peGFP-C1 *plasmid DNA solution is inserted into the YS cavity from the node region and through the amnios. Fast Green (0.01%) is added to the solution to visualise construct delivery. This injection mode, designed to limit plasmid release from the YS cavity, leads to the collapse of the amnion towards the ectoplacental cavity. Upon mouth driven-release of the plasmid, the amnion returns to its former position and an accurate injection can thus be visualized by the fast green filling of the YS cavity (Fig. 2C, D).

###### b-Electrode positioning and electroporation parameters

Electroporation allows gene delivery into the specified cell subset provided that the path of the negatively charged DNA towards the positive electrode (anode) is correctly adjusted. Careful examination of wholemount β H1 and lmo2 *in situ *hybridization patterns allocated the blood islands to the median third of the distance extending from the floor to the roof of the YS-cavity. Following injection, the two electrodes (gold genetrodes 512 from BTX) are thus positioned at each side of the YS (Fig. 2E), parallel to the presumptive blood islands ring.

**Figure 3:**
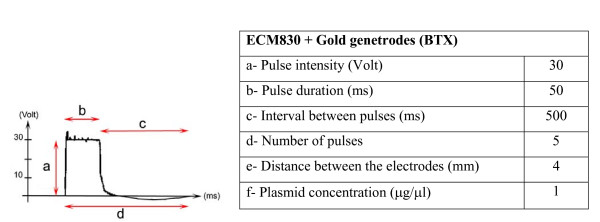
***In situ *electroporation parameters**. Left: Scheme of a square wave pulse delivered during *in situ *electroporation. Right: Optimal parameters used for YS *in situ *electroporation at the OB-EB stages.

We used a square-wave pulse generator (ECM830, BTX) to deliver the construct at a low voltage, thus preserving tissue integrity. The best results (efficient electroporation, preservation of explants morphology and recovery of a normal hematopoietic progeny from transfected precursors, see below) are obtained with five 50 ms pulses of 30 V at 500 ms intervals, with a 4 mm distance between the electrodes (Fig. 3). In theory, with such settings, a maximum 40–50% of the blood islands cells might take up the plasmid. In order to increase the targeted area, pulses were applied twice, with an inverted field, using the same parameter set-up. Unfortunately, the gain in the number of transduced cells was annihilated by a decrease in cell viability (Data not shown).

##### 3-Development of electroporated YS-explants during organ culture

**Figure 4:**
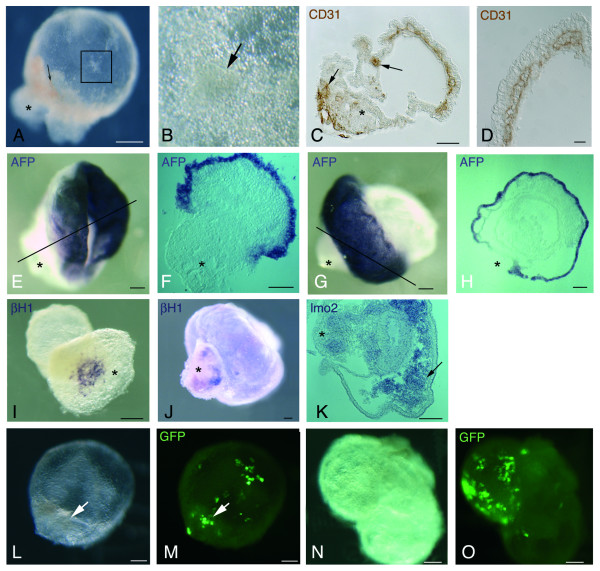
**Cytological analysis of electroporated OrgD3 YS-explants**. Abbreviations: AFP: α-foeto-protein; Control non-electroporated YS-explants (A), maintained in organ culture, organise into a "bubble-like" structure that contains a compacted part, at the site adhering to the culture dish (asterisk). Erythroid cells (arrow), differentiated from explanted YS mesoderm, are located close to this adhering site (A). The "bubble-like" part harbours clusters similar to YS-blood islands (B: Enlargement of the square in A), which also contains erythroid cells (arrow). CD31^+ ^cells are present in the both in the "bubble-like" structure and adhering site (asterisk) (C). Whereas in the adhering site, the nature of labelled cells (arrows) is unclear, CD31^+ ^endothelial cells (D) are clearly present cells in the bubble. In electroporated YS explants, both without (E, F) or with (G, H) plasmid, the endoderm, revealed by AFP wholemount *in situ *hybridization, remains external, but does not cover the whole mesoderm, as shown on the sections of wholemount embryos (F, H) made along the axis shown in E, G. β-H1^+ ^erythroid cells (I, control YS and J, electroporated YS) are mostly located at the adhering site (asterisk), while lmo2-expressing cells (K, control YS) are distributed either in the adhering site (asterisk) or in close contact with the endoderm (arrow). At the end of the organ culture step, both the blood islands-like clusters and the adhering sites (L, N) display GFP^+ ^cells (M, O: same explants as L, N), often in area containing erythroid cells (arrows). Scale bars: 100 μm, except D: 20 μm.

When placed in organ culture, YS-explants, both non-electroporated control and electroporated (with or without plasmid), rapidly form a bubble-like (Fig. 4A) structure that mimics YS development. It contains cell clusters similar to those observed in control blood islands (Fig. 4B). Moreover, differentiation along the erythroid (Fig. 4A, B) and endothelial (Fig. 4C, D) lineages normally occurs. After 3 days in organ culture, the explants have progressively organized into a bipolar structure with most mesoderm slightly adhering to the plastic dish, while the endoderm is exposed to the external environment, as shown by the expression of α-foeto-protein (AFP) transcripts, as a visceral endoderm marker [22] (Fig. 4E–H). Within the mesodermal layer, β-H1-expressing erythroid cells are mostly located at the adhering site (Fig. 4I, J see also Fig. 4A, L), while lmo2-expressing cells are more largely distributed in the YS-explants (Fig. 4K).

GFP can be visualized in electroporated YS after on average three hours and is still detected after the 3 days of organ culture. In YS explants, GFP^+ ^cells are mostly located within the blood islands-like clusters and the adhering site (Fig. 4L–O), which provides a first indication that the prospective blood islands has been accurately targeted.

##### 4-Hematopoietic recovery from electroporated YS-explants kept 3 days in organ culture

###### a-Characterization of the hematopoietic progeny of transduced cells

**Figure 5:**
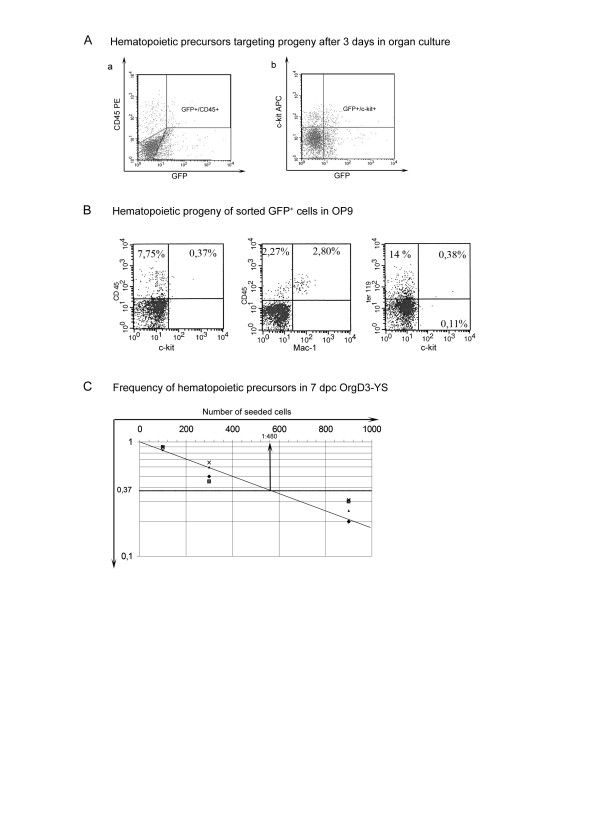
A: Hematopoietic progeny obtained from OrgD3 electroporated YS. GFP^+ ^cells display hematopoietic markers indicating that pre-hematopoietic mesodermal cells were targeted. B: GFP^+ ^cells sorted from electroporated OrgD3-YS give rise, after a 5 day culture on OP9 stroma, to the hematopoietic progeny typical of the YS, i.e. few precursors (left panel), myeloid (middle panel) and erythroid (right panel) cells. C: Frequency of hematopoietic precursors within OrgD3 control YS (see also Table 1) obtained by limiting dilution assay.

We next analysed by flow cytometry the presence of transduced hematopoietic cells in YS-explants immediately after the three days in organ culture (thereafter referred to as OrgD3-YS). The percentage of GFP positive cells represents on average 0,5–1% of the whole OrgD3-YS population (corresponding to 50–100 cells), a percentage classically obtained using unilateral electroporation procedure in mouse embryonic tissues [9,23]. The presence of both c-kit and CD45 positive cells within the GFP^+ ^subset (Fig. 5A) confirms that mesodermal/pre-hematopoietic cells, capable to give rise to a hematopoietic progeny, were successfully transduced.

In order to assess whether the targeted mesodermal/pre-hematopoietic precursors keep a normal differentiation pathway, we next analysed the differentiation potential of sorted GFP^+ ^cells after a 5 days culture on OP9 stromal cells. These cells gave rise to the typical YS progeny, namely Mac-1^+^/CD45^+ ^macrophages and Ter119^+ ^erythroid cells, and few c-kit^+^/CD45^+ ^precursors (Fig. 5B), indicating that the GFP plasmid was indeed transferred to mesodermal/pre-hematopoietic precursors. Interestingly, GFP is not expressed by their hematopoietic progeny (data not shown), reflecting the non-integrated status, and hence the transient expression, of the transgene.

###### b-Viability of electroporated hematopoietic precursors

The frequency of hematopoietic precursors present in control OrgD3-YS, as well as in OrgD3-YS electroporated without plasmid, was quantified by culture in limiting dilution on OP9 stromal cells for 5 days. In control OrgD3-YS, an average 1 out of 480 cells gives rise to a hematopoietic progeny, which corresponds to about 10–11 precursors per YS (Fig. 5C, Table 1). A similar frequency (1 hematopoietic precursor out of 453 cells) is obtained from OrgD3-YS electroporated without plasmid (Table 1), indicating that our electroporation protocol does not affect the recovery/viability of hematopoietic precursors.

**Table 1:** *In situ*electroporation efficiently delivers the construct to the blood islands

	**Frequency of recovered hematopoietic precursors**	**Number of recovered hematopoietic precursors per YS**	**Number of experiments**
**Whole OrgD3-YS**	1/475	10–11	4
**Whole OrgD3-YS electroporated without plasmid**	1/453	10	3
**OrgD3-YS GFP**^+^**cells**	1/150 (± 57.62)	0.56 ± 0.18 per 0.5 YS (targeted area)	5

Hematopoietic precursor frequency within whole control YS (electroporated without construct) and GFP^+ ^cells sorted from electroporated YS-explants were determined through limiting dilution assay.

###### c-Efficiency of the gene transfer into mesodermal/pre-hematopoietic precursors

We next quantified the recovery of hematopoietic precursors derived from transduced mesodermal cells. As stated above, our unilateral electroporation procedure only delivers the construct to at the best 40–50% of the blood islands, corresponding to 5–6 precursors per YS.

Sorted GFP^+ ^cells were distributed at 10 or 30 cells per wells on OP9 stromal cells and scored for the presence of a hematopoietic progeny five days later. In five independent experiments, on average 1/150 GFP^+ ^cell displayed a hematopoietic potential (Table 1). This value corresponds to a 3-fold enrichment in hematopoietic precursors compared to control OrgD3-YS. This enrichment indicates the presumptive blood islands were accurately targeted during the *in situ *electroporation step. However, the absolute number of GFP^+ ^hematopoietic precursors (0.56 ± 0.18 obtained per 0.5 YS-equivalent) remains low (Table 1), even though a hematopoietic progeny is systematically obtained from transduced mesodermal/pre-hematopoietic precursors.

### B-Transduction of 8 dpc YS-hematopoietic precursors

#### I-*In situ *electroporation

We investigated whether *in situ *electroporation is also efficient to target hematopoietic precursors at later development stages (From EHF stage to the 5-S stage). The same settings were applied to transfer the plasmid to the blood islands. Electroporated YS were kept in organ culture for one day (instead of the 3 days in organ culture for 7 dpc YS, required for the differentiation of mesodermal/pre-hematopoietic precursors) to allow for GFP expression prior to sorting. Flow cytometry analyses, performed immediately after the organ culture step, showed that GFP-expressing cells again represented 0.5–1% of the whole population. Upon sorting and culture on OP9 stromal cells for 5 days, these cells produced a hematopoietic progeny comparable to that obtained from whole control (non-electroporated) YS. These data indicate that *in situ *electroporation may be used to transduce hematopoietic precursors at 8 dpc.

#### II-Retroviral transduction

However, at this stage, the blood islands have developed into vessels filled with hematopoietic cells, so that the hematopoietic precursors are diluted amongst erythroid cells (Figure 1D, H). Thus electroporation appears less convenient at this stage while a method ensuring a high efficiency of transduction (with respect to the whole population) would be more adapted. In addition, at 8 dpc, contrary to 7 dpc, blood islands precursors can be directly obtained from dissociated YS. We took advantage of these features to examine whether retroviral transduction would be adapted to efficiently transfer genes into hematopoietic precursors from 8 dpc YS.

##### 1-General scheme of the protocol

Transduction using ecotropic retrovirus has previously been used to transfer HoxB4 into 8 day YS hematopoietic precursors [13]. However, neither quantitative data nor optimization of the various infection parameters was provided.

As a first step, the following protocol was designed to target gene to hematopoietic precursors from 8 dpc (0–4 somite stages) YS. In this study, transduction of hematopoietic precursors was obtained using a MSCV-based retroviral vector called MPI. This retroviral vector harbours, as a FACS-selectable reporter, the *eGFP *cDNA downstream a IRES element [24].

We first compared the transduction efficiency attained using freshly isolated YS or YS explants dissociated after one day in organ culture (thereafter referred to as OrgD1-YS). In both conditions, we systematically analysed: 1-GFP level at day 1 post-infection (*i.e*. 24 hours after exposure to the viral supernatant), 2- the persistence of GFP expression in YS-transduced cells during *in vitro *hematopoietic differentiation, 3- the viability and phenotype of the hematopoietic progeny of sorted GFP^+ ^cells, 4- the number and the type of hematopoietic cells produced upon *in vitro *culture on OP9 stromal cells or in methylcellulose assay. In both instances (8 dpc YS freshly explanted or maintained in organ culture for one day), all these analyses were performed after GFP^+ ^cell sorting at day 1 post-infection.

##### 2-Retroviral transduction of 8 dpc YS on freshly isolated cells or after organ culture

**Figure 6:**
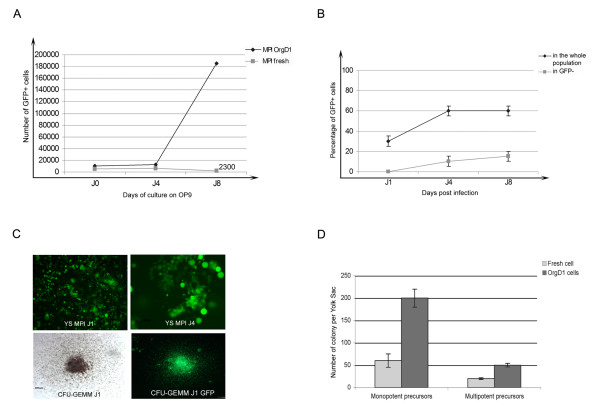
A: Compared evolution of cell production obtained from GFP^+ ^cell sorted from 8 dpc YS infected upon explantation (between bracket: number of cells recovered at day 8), or after one day in organ culture. B: Evolution of GFP expression in the whole population or in GFP^- ^cells sorted from transduced 8 dpc OrgD1-YS after culture on OP9 stromal cells. A steady state GFP expression is attained at day 4 post-infection. Moreover, GFP expression is acquired, during culture, by a subset of GFP^- ^sorted cells. C: GFP expression in transduced OrgD1-YS cells (top panel left) persists in sorted GFP^+ ^cells cultured on OP9 stromal cells (top panel right). The colonies generated by transduced cells also remain entirely GFP^+ ^(bottom panel).

In our first tests, we used the MPI retroviral vector to transduce 8 dpc YS directly upon dissection. After dissociation, YS cells are seeded in a 48-well plate in 1 ml medium supplemented with cytokines and 4 μg polybrene (see Materials and Methods). After addition of the retroviral supernatant (MOI = 1), the culture is maintained at 37°C, 5% CO_2 _for 12 hours and re-plated in fresh medium and either seeded on OP9 stromal cells or analysed for clonogenic potential in methylcellulose assay. In these conditions, the initial efficiency of transduction is reproducible and quantitatively high (60–70% GFP^+ ^cells). However, the GFP^+ ^population rapidly decreases in culture to completely vanish after 4–5 days (Fig. 6A). The 8 dpc YS is enriched in terminally differentiated hematopoietic cells and immature erythro-myeloid precursors only appear at the 2–5 somite-stage [25,26]. Accordingly, we suspect that the rapid exhaustion of the GFP^+ ^population during the 4 days culture arises as a consequence of the relative maturity of the transduced cells, which therefore rapidly complete differentiation and disappear.

A mean to obtain a larger number of targeted hematopoietic precursors and still avoid cross-contamination by intra-embryonic-derived hematopoietic precursors, is to place the YS, dissected before the 4 somite-stage for one day in organ culture (OrgD1), as previously described [3,4]. Thus, dissociated OrgD1-YS cells were exposed to the retroviral supernatant with the same MOI and cultured as described above. Under these conditions, transduction efficiency is also highly reproducible, but lower (25–30%) than in the former conditions. However, in sharp contrast with the former conditions, the absolute number of GFP^+ ^cells increases between 4 and 8 days post-infection (Fig. 6A). Moreover, the percentage of GFP^+ ^cells also increases during the culture to reach a maximum (50–60%) at day 4 post-infection (Fig. 6B). Since about 15–20% of GFP-negative cells sorted at day 1 post-infection are shown to acquire GFP expression over the following days in culture (Fig. 6B), the increase in GFP^+ ^population between day 1 and day 4 post-infection may result, at least in part, from *de novo *GFP expression. Interestingly, when observed in semi-solid culture, the colonies appear as entirely composed of strongly GFP positive cells, which indicates that the whole progeny of transduced hematopoietic precursors keeps the expression of the transgene (Fig. 6C). The threefold increase in the number of monopotent as well as multipotent clonogenic precursors among the GFP^+ ^cells at day 4 compared to that obtained from 8 dpc YS transduced immediately after explantation (Fig. 6D), confirms that inclusion of a organ culture step leads to a transduction of a higher number of hematopoietic precursors. The persistence of GFP expressing cells, when transduced cells derive from OrgD1-YS, thus reflects that the organ culture step indeed improves the targeting of immature precursors.

We therefore focused on the protocol including the organ culture step for further analyses of the hematopoietic phenotype and differentiation potential of transduced cells.

##### 3-Hematopoietic development from YS transduced cells

Flow cytometry analysis of the percentage of 7AAD labelled cells shows no difference in cell viability between infected or non infected YS-cells after one or four days in culture on OP9 stromal cells (Data not shown). In our conditions, the proliferation rate is perfectly equal between transduced and non-transduced cells (Data not shown).

**Figure 7:**
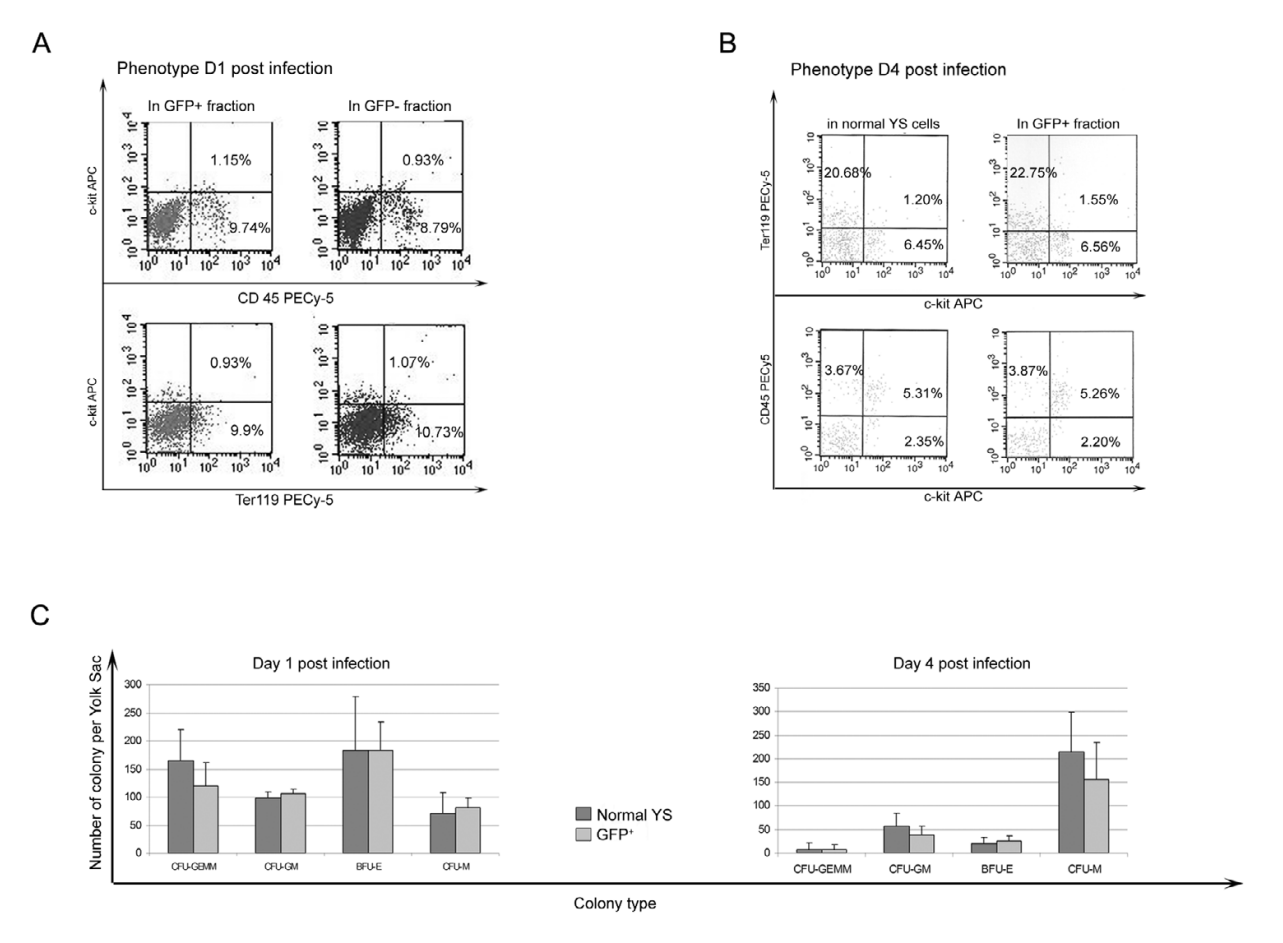
**Hematopoietic development from transduced cells**. A: Comparison of the clonogenic potential of GFP^+ ^cells sorted at day 4 post-infection from YS infected upon explantation or after one day in organ culture. B: Phenotype analyses of OrgD1-YS infected cells cultured for 1 day on OP9 stromal cells, show that erythroid (Ter199^+^) and myeloid (CD45^+^) cells are identically distributed within GFP^+ ^and GFP^- ^subsets. C: At day 4 post infection, the distribution of erythroid (Ter199^+^) and myeloid (CD45^+^) cells within the GFP^+ ^population is similar to that obtained from normal YS (never exposed to viral supernatant). D: Clonogenic assays, performed at day 1 (left panel) and 4 (right panel) post-infection, indicate that the transduction procedure does not modify the type and numbers of precursors recovered.

The phenotype of hematopoietic cells recovered from OrgD1-YS at 24 hours post-infection is similar to that of control OrgD1-YS (data not shown). Moreover, the various hematopoietic subsets, both myeloid (CD45^+^) and erythroid (c-kit^+^/Ter 119^+^) are similarly distributed within the GFP^+ ^and GFP^- ^populations (Fig. 7A). The fact that the retrovirus-mediated transduction protocol does not disturb hematopoietic differentiation from OrgD1-YS is also evidenced upon phenotype analyses performed at day 4 after culture on OP9 stromal cells, since the relative percentage of erythroid and myeloid cells is not modified in transduced cells compared to normal YS (Fig. 7B).

We coupled the phenotype analyses to *in vitro *clonogenic assay to verify that the differentiation potential of transduced precursors was not modified by the whole procedure. As shown in Fig. 7C, in three independent experiments, the clonogenic potential obtained from sorted GFP^+ ^does not significantly differ from that obtained from similar number of OrgD1 control (normal) YS, both at days 1 and 4 post-infection. The clonogenic potential thus correlates with the phenotype analyses performed at the same time points (Fig. 7A,B).

In conclusion, there is no phenotypic as well as differentiation potential bias between GFP^+ ^and GFP^- ^cells recovered from Org-D1 transduced YS. Moreover, our results (to be published elsewhere) obtained following retroviral transduction of a candidate gene into Org-D1 YS indicates that insertion of a 2 kb cDNA into the MPI vector: 1- does reduce neither the GFP level nor the percentage of transduced cells, 2- allows the identification of the exogenous protein through Western Blot. This procedure is thus adapted to analyse the effect of candidate gene on YS-hematopoietic cells development.

## Discussion

The analysis of the first steps of hematopoietic development historically performed though the study of the yolk sac, is somehow limited by the poor level of available precursors, as well as their extremely fast differentiation. Great progress in the understanding of the development of primitive hematopoiesis was achieved, owing to the ES model, once the similarity of developmental sequence with that which occurs *in vivo *was firmly established [27]. The availability of large number of ES cells, amenable to genetic manipulations, further refined our knowledge of the mechanisms involved (e.g. through invalidated ES cells rescue experiments). Unfortunately, the gathered information cannot be easily placed back to back to similar experiments conducted in the "normal" YS developmental context.

Here we provide two gene transfer protocols, whose combination may allow a thorough study of both early and late events of hematopoietic development in the YS. Thanks to two markers which make it possible to discriminate between mesodermal/pre-hematopoietic (lmo2^+^/β-H1^-^) [17] and their erythroid progeny (lmo2^+^/β-H1^+^), we precisely determined the stage when the YS blood islands are enriched in immature precursors. This expression analysis also allowed to precisely locate the prospective blood islands in intact 7 dpc YS. We next used this delineation to perform *in situ *electroporation, which keeps intact the mesoderm-endoderm interactions shown to be crucial for proper hematopoietic development at these stages [28,29]. This approach was proven here to be effective to target mesodermal/pre-hematopoietic precursors and to be completely compatible with a normal viability and hematopoieisis of the transfected mesodermal/pre-hematopoietic precursors upon organ culture and subsequent *in vitro *culture on OP9 stromal cells. Thus, our work provides for the first time a detailed procedure allowing the analysis of the very early steps (determination) of primitive hematopoiesis. However, although our protocol ensures a significant enrichment of hematopoietic cells in the transfected population (three folds), the overall yield remains low (about 1 transfected mesodermal/pre-hematopoietic precursors per YS) for two reasons: 1- the intrinsic paucity of this precursor type in the 7.5 YS (about 10, after the 3 days in organ culture); 2- the relatively low, albeit usual in related experiments [9,23], level of gene transfer through *in situ *electroporation. Yet, this limitation does not disqualify the procedure since qualitative analyses following transfer of candidate genes can be performed.

A complementary protocol was then provided to transfer gene in « late » (8 dpc) YS which contains numerous determined cells. At this stage, hematopoietic precursors are much less precisely localized than their mesodermal ancestors, but rather dispersed and diluted among an abundant differentiated (essentially erythroid) progeny. Moreover, the differentiation of these cells is not anymore dependent upon interaction with endodermal cells. We thus reasoned that a « blind » (i.e. non selective) but efficient method would be the most adapted and therefore performed ecotropic retrovirus-mediated gene transfer onto isolated YS cells. A similar strategy has been previously used to express HoxB4 into YS cells at about the same stage [13]. However, neither indication about the efficiency of the method, nor quantitative data were provided. This is of particular importance given that the nature of the transduced gene in this study may confer such a high competitive growth/survival advantage that even a weak efficiency of transduction may have been sufficient. We show here that even for a «neutral» gene (*eGFP*), the transfer through ecotropic retrovirus in 8 dpc YS hematopoietic precursors is very potent, therefore overcoming the problem of precursor dilution. Indeed, over 60% of the YS cells were transduced using a MOI as low as 1. In fact, YS cells appear highly susceptible to retroviral transduction as a very short contact (4–6 hours) with the viral supernatant is sufficient to produce a maximal transduction which therefore minimizes the potential side effects of concentrated viral supernatant or of polybrene, and allows a precise temporal targeting of the beginning of gene transfer. Moreover, we show that including a 24 h organ culture step before exposure to the viral supernatant markedly improves the targeting of immature precursors. This may result from an increased precursor frequency, as evidenced by the higher recovery of clonogenic precursors obtained when GFP^+ ^cells are sorted from OrgD1-YS. As a consequence, even though the protocol that includes an organ culture step leads to a lower percentage of transduced cells, GFP^+ ^cells steadily increase from day 4 post-infection, while they rapidly vanish when recovered from freshly transduced YS. Further analysis of the hematopoietic progeny of transduced cells is therefore easier under these conditions. Confirming the high permissiveness of YS cells to retroviral transduction, we found that a MPI vector carrying a 2 kb cDNA is almost as efficient as the empty one at transducing OrgD1-YS hematopoietic precursors.

As for *in situ *electroporation of 7 dpc YS, the recovery, survival and differentiation potentials of retrovirally transduced YS cells appear undistinguishable from that of non-transduced cells. Thus, retroviral transduction appears as a very convenient way to genetically modify hematopoietic precursors from 8 dpc YS.

Two potential problems, however, limit the use of retroviral transduction:

1- in our conditions, 7 dpc YS cells (i.e. containing mesodermal/pre-hematopoietic precursors cells), which are amenable to gene transfer by *in situ *electroporation, appear almost totally refractory to retroviral transduction. The reason for this pitfall is unknown, but we suspect that, at this early development stage, mesodermal cells have not yet reached some degree of commitment/differentiation that allows retroviral transduction. Indeed, 7 dpc YS mesodermal/pre-hematopoietic precursors become efficiently transduced after a two days of organ culture, which essentially recapitulates their *in vivo *progression toward committed precursors.

2- Retroviral vector are stably integrated, and therefore transduced cells are indelibly genetically modified. This is confirmed by the expression of GFP after at least two weeks of culture (latest time point analysed) in retrovirally transduced cells from late YS. In contrast while GFP is still detectable in electroporated cells from 7 dpc YS after 4 days, it completely vanished a few days later. In view of this limitation, we show that our *in situ *electroporation protocol transduce hematopoietic precursors from 8 dpc YS and thus deserves to be used notably if a transient expression is needed. Note that a 4 days expression may appear somewhat long with respect to the accelerated dynamic of embryonic events, especially in the case of early hematopoiesis. However, given the intense proliferation of early YS cells, we suspect that the plasmid delivered by *in situ *electroporation is in fact rapidly diluted so that the expression of labile proteins will be expected to be more really « transient » than that of GFP, whose half life exceeds 24 hours.

## Conclusion

In summary (Table 2), we provide and detail here two complementary methods to transfer genes in YS cells, at the earliest stages of hematopoietic development (7–8 dpc). We studied the influence of several parameters and hence determined the most convenient for either early (mesodermal/pre-hematopoietic) and late (hematopoietic) precursor transduction. These two methods can be used to either enforce (as exemplified here) or knock-down (through vectorized siRNA) the expression of potentially important genes. They are thus likely to increase our knowledge of the mechanisms giving rise to primitive hematopoiesis and to understand the molecular basis underlying its limited potency, when compared to true HSC from the AGM or adult BM.

**Table 2:** Assets and limitation of *in situ *electroporation and retroviral transduction to target YS blood islands precursors

	*In situ *electroporation		Retroviral transduction	
		
	Asset	Limitation	Asset	Limitation
7–7.5 dpc: Mesodermal/pre-hematopoietic precursors	- Transient - Layer specific delivery - Spatial control of delivery	- Reduced number of precursors available for transduction		- Causes extensive cell death - Refractory to transduction ?

Application	Only possible method		Unsuitable at this development stage	

From 7.5–8 dpc: Hematopoietic precursors	- Transient - Layer specific delivery - Spatial control of delivery	- Only a fraction of the YS blood islands is targeted	- Easy - All hematopoietic precursors may be transduced	- Does not allow layer specific targeting

Application	-Transient transgene delivery		- Sustained expression of the transgene	
	- Possible layer specific delivery.		- Possible delivery of ShRNA	
	- Possible delivery of SiRNA or proteins			

Hematopoietic precursor frequency within whole control YS (electroporated without construct) and GFP^+ ^cells sorted from electroporated YS-explants were determined through limiting dilution assay.

## Methods

### 1-Mice and isolation of embryo

All animal experiments were conducted in compliance with French and European regulations. Balb/c or C57BL/6 males are crossed with females to generate embryos and the morning of the vaginal plug observation is considered as 0.5 dpc. Pregnant females are sacrificed by cervical dislocation. Embryos at 8 dpc are staged by somite counting. Presomitic embryos are staged according to the development of the allantois bud: OB (No Bud), EB (Early Bud) and LB (Late Bud) [30].

### 2-Isolation and organ culture of 7 and 8 dpc Yolk Sacs

Embryos from OB to EB stages, used for *in situ *electroporation, are collected in Hanks balanced salt solution (HBSS: Invitrogen), and after removal of the Reichert membrane (the ectoplacental cone being preserved), they are injected with the plasmid solution as described below. After injection and pulse application, the extra-embryonic compartment is removed with ultra-fine forceps and maintained in organ culture as described below. From 8 dpc, the YS used for retroviral infection, are directly explanted from the embryos.

YS explants are transferred into 6-well plates (TPP) containing "Complemented OptiMEM medium", i.e. OptiMEM with Glutamax (Invitrogen), 10% Fetal Calf Serum (Hyclone), 1% Penicillin-steptomycin (Invitrogen) and 0.1% β-mercaptoethanol (Invitrogen), and are kept in culture at 37°C, 5% CO_2 _for 1 (8 dpc) or 3 (OB and EB stages) days, before analyse (OB and EB stages) or retroviral infection (8 dpc). Prior to further processing, YS-explants are dissociated by gentle pipeting and filtered to obtain a single cell suspension.

### 3-*In situ *Electoporation

For *in situ *electroporation, we use the peGFP-C1 vector (Clontech), in which the cytomegalovirus (CMV) promoter drives the expression of the GFP reporter protein.

To visualise plasmid solution delivery, 0.01% Fast Green is added to 1 μg/μl *peGFP-C1 *plasmid DNA. Borosilicate standard wall (1.0 mm O.D./0.58 mm I.D.) glass capillaries (Harvard Apparatus) are pulled and filled with DNA/Fast Green solution for injection.

After dissection, the plasmid solution is injected in the YS cavity as described in Fig. 2, and embryos are placed in a Petri dish, in HBSS. To accurately target the presumptive blood islands, OB-EB embryos are oriented so that the caudal half part of the visceral YS is facing the anode. The two gold genetrodes (BTX Instrument Division Ref 512) are positioned parallel to the presumptive blood islands, and pulses are applied using a square-wave pulse generator (Electro Square Porator ECM 830, BTX Instrument Division; San Diego, CA, USA).

### 4-Retroviral infection of 8 dpc YS

#### Production of ecotropic retroviral supernatants

We used for retroviral transduction a MSCV-based retroviral vector, called MPI [24]. HEK 293T cells (4 × 10^6 ^cells; 30–40% confluence) are seeded in DMEM supplemented with 10% FCS and 1% Penicillin-steptomycin (Invitrogen), in 150 mm culture dishes. The following day, the MPI expression vector (6.4 μg) is co-transfected with 3.2 μg phCMV-intron GagPol and 3.2 μg FB-MO-SALF [31] helper plasmids, encoding the MLV gag-pol and ecotropic env proteins, respectively, using Fugene 6 (Roche), as transfection reagent, to produce retroviral particles. Viral supernatants are collected twice a day, from 24 h to 72 h post-transfection, filtered (0.45 μm) and concentrated on Centrifugal Filter Devices (Centricon^®^Plus-20 from MICON Bioseparation). Concentrated supernatants are distributed into 30 μl aliquots, kept frozen at -80°C. Titration is performed by transducing mouse 3T3 fibroblasts (plated at 10^5 ^cells per well in 6-wells plates) in exponential growth phase with 5 μl concentrated supernatant in the presence of 4 μg/ml polybrene. Cells are analysed for GFP expression 4 days post exposition to the viral supernatant, by flow cytometry using a Facscan (Becton Dickinson). Typically, the titre of concentrated supernatants is 7 × 10^6^-10^7 ^viral particles per ml.

#### Retroviral transduction conditions

Ten dissociated YS explants (about 10^5 ^cells) are seeded in 48 well-plates containing 1 ml "Complemented OptiMEM medium", further supplemented with Flt3-ligand (20 ng/ml), Stem Cell Factor (100 ng/ml), EPO (10^3 ^U/ml) and 4 μg polybrene. Viral supernatant is added to obtain one retroviral particle per cell (MOI = 1) during 12 hours. After infection, transduced cells are transferred on OP9 stromal cells and cultured as described below. Interestingly, a 25–30% transduction efficiency is achieved on OrgD1-YS, either with a 24 hours or a shorter (4 or 6 hours) exposure to the retroviral supernatant.

### 5-Expression analyses

#### *In situ *hybridization

YS explants, fixed in 4% paraformaldehyde in PBS overnight at 4°C and rinsed with PBS, are hybridized with riboprobes at 65°C, as previously described [32]. Digoxigenin (DIG)-labelled antisense RNA probes are obtained from PCR fragments subcloned into expression vectors (βH1, provided by I. Max-Audit: 250 bp subcloned into pCR™; Lmo-2 constructed by V. Lemarchandel: 450 bp subcloned into pBSKII and α-foeto-protein (AFP) provided by L. Robb: 845 bp subcloned into pBSKII) and synthesized using the DIG RNA Labelling Kit (Roche Diagnostics, Indianapolis, IN). To obtain cryostat sections of hybridized samples, YS explants are fixed (4% paraformaldehyde, 4% sucrose, 0.12 M CaCl_2_, 0.2 M Na_2_HPO_4_, 0.2 M NaH_2_PO_4_, H_2_O), embedded in 0.12 M phosphate buffer, 7.5% gelatin, 15% saccharose and frozen in liquid nitrogen. Embedded embryos are sectioned at 10–15 μm and transferred to Superfrost slides.

#### Immuno-histochemistry

Cryostat sections of YS explants, prepared as described above, are incubated first with 0.3% H_2_O_2 _to block endogenous peroxydase activity, then in PBS, 10% fetal calf serum (2 hours at room temperature) to prevent non specific antibody binding. Samples are incubated with purified Rat anti-Mouse CD31 (clone MEC13.3, Pharmingen) overnight at 4°C, followed by incubation with biotinylated-Goat anti-Rat (DAKO), as secondary antibody for 1 hour at room temperature and Streptavidine-horseradish peroxydase complex (VECTOR) for 30 Min. at 37°. Peroxydase activity is revealed by exposure to its diaminobenzidine substrate. Sections are mounted in Glycergel (DAKO) and photographed using DIC optics.

### 6-Microscopy and photography

Samples are observed either on a Fluorescence-equipped Olympus SZX12 stereomicroscope (wholemount *in situ *hybridized or electroporated YS) or on a Zeiss Axioplan microscope (YS-explants sections). Images are acquired respectively with the DP50 Olympus digital camera (Analysis software) or Zeiss AxioCam MRc (Axiovision 4.3 software). Images are further processed with the Adobe Photoshop CS software.

### 7-Analysis of hematopoietic cell production

#### Culture on OP9 stromal layer

Cells are seeded on OP9 stromal cells (from S.-I. Nishikawa and T. Nakano, Riken Center for Developmental Biology, Kobe, Japan), in "Complemented OptiMEM medium", further supplemented with Flt3-ligand, Stem Cell Factor and EPO, and cultured as described in [32]. At various culture time points, cells are analysed by flow cytometry or submitted to clonogenic assays.

#### Clonogenic assay

Cells recovered after 1 or 4 days from the culture on OP9 stroma, are assayed for the presence of colony-forming units, by seeding 3000 cells per 30 mm culture dish, in triplicate, in 1 ml methylcellulose medium (M3234; Stem Cell Technologies, Vancouver, BC, Canada) supplemented with cytokines (50 ng/ml Stem Cell Factor, 10 ng/ml Il-3, 10 ng/ml Il-6, 3 U/ml EPO and 10 ng/ml TPO). After 3 to 7 days at 37°C, colonies are scored based on their morphology on an inverted microscope.

#### Flow Cytometry Analyses and cell sorting

Cells are analyzed on a FACS Sort (Becton-Dickinson) as previously described [32], and FACS data are analyzed with CellQuestPro software (Becton-Dickinson).

The following antibodies (all from Pharmingen) are used to reveal the presence of lineage-specific surface markers: CD45 (Clone 30-F11), CD31 (Clone MEC 13.3) and Mac-1 (Clone M1/70) for myeloid cells, Ter119 (Clone Ly-76) for erythrocytes. Cell sorting was performed with a FACS Diva cell sorter (Becton-Dickinson).

## Abbreviations

AGM: Aorta-Gonad-Mesonephros; EB: Early allantoic Bud stage; LB: Late allantoic Bud stage; LHF: Late Head Fold stage; HSC: Hematopoietic Stem Cell; LTR: Long Term Reconstitution (capacity); OB: No allantoic Bud stage; Sp: Splanchnopleura; YS: Yolk Sac.

## Competing interests

The author(s) declare that they have no competing interest.

## Authors' contributions

IG conceived the project. IG and OA supervised the project. IG, OA and SG designed experiments, analysed the results and wrote the manuscript. PM assisted in experimental design. SG carried out most of the experimental work. CA-L and YL participated significantly to data acquisition. All authors read and approved the final manuscript.
